# Outreach-based clinical pharmacist prescribing input into the healthcare of people experiencing homelessness: a qualitative investigation

**DOI:** 10.1186/s12913-020-06013-8

**Published:** 2021-01-04

**Authors:** Sarah Johnsen, Fiona Cuthill, Janice Blenkinsopp

**Affiliations:** 1 Institute for Social Policy, Housing and Equalities Research, Heriot-Watt University, Edinburgh, UK; 2 School of Health in Social Science, University of Edinburgh, Edinburgh, UK

## Abstract

**Background:**

Severely and multiply disadvantaged members of the homeless population are disproportionately vulnerable to exceptionally high levels of multi-morbidity and premature death. Given widespread calls for the development of interventions that might improve the uptake and effectiveness of healthcare for this population, this study investigated patient and other stakeholder perspectives regarding an outreach service, delivered by prescribing pharmacists in collaboration with a local voluntary sector provider, within homelessness services and on the street in Glasgow (UK).

**Methods:**

The qualitative study involved semi-structured face-to-face interviews with 40 purposively sampled individuals with current or recent experience of homelessness (32 of whom had direct experience of the service and 8 of whom did not), all (*n* = 4) staff involved in frontline delivery of the service, and 10 representatives of stakeholder agencies working in partnership with the service and/or with the same client group. Pseudonymised verbatim interview transcriptions were analysed systematically via thematic and framework analysis.

**Results:**

The service was effective at case finding and engaging with patients who were reluctant to utilise or physically unable to access existing (mainstream or specialist ‘homeless’) healthcare provision. It helped patients overcome many of the barriers that homeless people commonly face when attempting to access healthcare, enabled immediate diagnosis and prescription of medication, and catalysed and capitalised on windows of opportunity when patients were motivated to address healthcare needs. A number of improvements in health outcomes, including but not limited to medication adherence, were also reported.

**Conclusions:**

A proactive, informal, flexible, holistic and person-centred outreach service delivered within homelessness service settings and on the street can act as a valuable bridge to both primary and secondary healthcare for people experiencing homelessness who would otherwise ‘fall through the gaps’ of provision. Prescribing pharmacist input coupled with third sector involvement into healthcare for this vulnerable population allows for the prompt treatment of and/or prescription for a range of conditions, and offers substantial potential for improving health-related outcomes.

**Supplementary Information:**

The online version contains supplementary material available at 10.1186/s12913-020-06013-8.

## Background

### Homeless healthcare context and challenges

The links between homelessness and poor health have been increasingly well documented internationally in recent years [1]. Existing evidence indicates that people experiencing homelessness are disproportionately vulnerable to premature death, and experience ill health at greater rates and at an earlier age than is true of the general population [2–4]. Higher mortality rates are generally attributed to acute and chronic medical conditions, substance misuse, violence, suicide or unintentional injury [3, 5–7]. The prevalence of tuberculosis, HIV, and hepatitis C is also higher amongst people experiencing homelessness [8, 9]. Evidence also suggests that levels of multi-morbidity are exceptionally high amongst the most ‘severely and multiply disadvantaged’ members of the homeless population who concurrently experience issues such as substance misuse and/or contact with the criminal justice system [10].

Existing literature also highlights the difficulties that healthcare and other support providers encounter in meeting the needs of multiply disadvantaged homeless people [11, 12]; so too the barriers and stigmatised attitudes often encountered by homeless people when attempting to access or use mainstream healthcare [13–15]. Particular concerns have been expressed regarding this population’s limited use of primary care services which contributes to increased use of acute emergency secondary care services during clinical crises [16]. It has also been shown that prescribing and adherence to medicines is lower for people experiencing homelessness as compared with housed populations [10, 17]. Furthermore, current evidence indicates that people experiencing homelessness rarely receive preventative care, but rather tend to access healthcare services only when sick or injured, or in need of medicine for pain or distress [18].

Against this backdrop, there is an increased recognition of the failure of mainstream services to meet the health-related and other needs of severely and multiply disadvantaged homeless people [11, 12]. Notably, a recent systematic review of inclusion health initiatives highlighted the value of a range of factors improving outcomes for vulnerable populations, including homeless people, such as: coordinated care, partnership working, service design which takes account of the whole person, service user involvement, active engagement, psychologically informed approaches, and provision of outreach in the community and on the streets [19]. Even so, and echoing gaps in evidence regarding ‘what works’ in addressing homelessness more broadly [20], there remains a paucity of evidence with respect to the effectiveness of interventions aiming to engage with and improve the health of people experiencing homelessness [21].

Policy makers and practitioners are increasingly calling for the development of interventions that might improve the uptake and effectiveness of healthcare for this population [22]. This paper documents the findings of a qualitative investigation of stakeholder perspectives and experiences of people receiving one such innovation. The Pharmacy Homeless Outreach Engagement Non medical Independent Rx (PHOENIx) service is delivered by a team of three pharmacists with an independent prescribing qualification in collaboration with a dedicated street team outreach worker from a third sector homelessness agency (Simon Community Scotland) in Glasgow (UK). The service has existed in some form for several years, but has recently evolved from being solely health clinic based to include outreach delivered in local homelessness services and on the street [23–25]. The centrality of pharmacist input makes this initiative distinctive from other mobile homeless health services, which (in the UK at least) tend to be led by nurses with input from General Practitioners (GPs) and/or other health professionals such as podiatrists or counsellors [26].

### Outreach-based pharmacist input

The PHOENIx service provides weekly ‘pop up, drop in’ outreach clinics at designated times within several local day centres, soup kitchens, an advice hub, homeless hostels, and bed and breakfast hotels (B&Bs) that are commonly used as temporary accommodation in the city. It also runs an established clinic within Glasgow’s specialist NHS Homeless Health Service (HHS) alongside the GPs. No formal appointment is required: patients either drop in on a first come, first served basis, or sign up with staff of the host agency beforehand. The service has also included an element of outreach on city centre streets since late 2017. During street outreach, potential patients are invited to participate in a health check and, if they consent, a venue (e.g. café) is found nearby in order that this may take place. The pharmacists work alone, with access to advice from the duty HHS GP if required and in conjunction with the GPs, when in clinics at the HHS. In all outreach contexts (e.g. hostels, day centres, and on the street), the team is accompanied by a trusted voluntary sector street team worker from Simon Community Scotland who proactively engages in informal conversations with the homeless people encountered, explains what the service can offer, builds relationships, and seeks to address any housing, welfare benefit and other issues identified.

In terms of initial assessment, if patients are willing, a full ‘health check’ is conducted using a tool which assesses several aspects of health: cardiac, respiratory, nutrition, addiction, psychological, feet, blood borne virus, and sexual health. This includes a mix of validated measures (e.g. EQ-5D5L, PHQ-4, CAGE) as well as closed and open-ended questions asking patients about various aspects their self-perceived health status and engagement with healthcare. Near patient tests assessing height, weight, blood pressure, blood glucose and pulse are used, and details of family history recorded. A review of medication is included. Primary and/or secondary healthcare records are accessed remotely when appropriate and patients grant permission. The information collected is used to formulate a plan together with the patient on priority issues to be addressed. If appropriate, conditions can be treated (e.g. wounds dressed) and/or a repeat or new prescription issued ‘then and there’.

As independent prescribers, PHOENIx service pharmacists can legally prescribe any medication within their competency [27]. In practice, they prescribe any routine primary care initiated medicines other than those used for the treatment of opiate dependency and alcohol withdrawal, because these are initiated and continued by alcohol and drug recovery services in Glasgow. The team aims to consult with patients repeatedly to monitor and facilitate delivery of care. Relevant information is forwarded to the patient’s GP after consultations, and referrals to GPs or other healthcare providers made as appropriate.

The analyses of PHOENIx patient records relating to samples of 124 and 52 individuals respectively reported by Lowrie R et al. [25] and Lowrie F et al. [23] provide a helpful overview of the type and nature of interventions delivered. Patients in the samples consisted primarily of white middle-aged Scottish men (mean ages recorded by the two studies were 40 and 45 years) who were living in homeless hostels, other temporary/insecure housing or sleeping rough. The most recent of these reports [23] indicates that medications were prescribed during consultations by the pharmacists to 62% of all patients; of these cases, new medications were initiated in 69%, repeat/re-issues of lapsed medications in 66%, and changes made to existing medication in 16%. The most commonly prescribed items included: wound dressings; antihypertensives; antidiabetics; analgesics; inhalers; antidepressants; and nutritional supplements. Pharmacists diagnosed a new clinical issue in 69% of patients, most commonly with infections (skin or respiratory) in 36% of patients. Nearly two thirds (62%) of patients had their presenting symptoms managed by the pharmacist alone. A total of 85% of patients subsequently attended either a follow-up with a pharmacist or onward referral to specialist services, secondary care, or a GP.

Glasgow is an apposite context in which to explore the implementation of such a service given recent reports highlighting the scale of rough sleeping [28], high level of multi-morbidity within the local homeless population [10], and number of deaths of homeless people in the city [29]. In policy terms, the need to ‘do things differently’ for the most excluded homeless people, and improve the links between housing and health provision in particular, are widely acknowledged at the national level [4, 28, 30, 31]. Parallel to this, and echoing developments in inclusion health more generally [19], strategic government publications in Scotland and England have recently advised that the pharmacy workforce increase its role in health promotion in an effort to enhance both capacity and capability in reducing health inequality amongst vulnerable populations [32, 33].

## Methods

### Sampling and data collection

The analysis described below is drawn from an independent evaluation of the PHOENIx service. The evaluation was underpinned by a realist methodological orientation which seeks to understand ‘what works for whom in what circumstances?’ [34]. Qualitative face-to-face, semi-structured individual interviews were conducted with three key groups. The first involved 40 individuals with current or recent *experience of homelessness* whom were purposively sampled to include a majority of patients who had used the service *(n = 32)* and a minority of individuals who had not used it *(n = 8).* The former were recruited to explore their individual experiences and perceptions of the service; the latter to seek their thoughts regarding what, if anything, might be done to encourage their use of it or healthcare more generally*.* Patients were purposively sampled so as to include insights from those who had accessed the service via a range of routes: 17 reported that they had first came into contact with it in a day centre, 12 in their temporary accommodation (hostel or B&B), two on the street, and one via the city’s specialist homeless healthcare service (HHS). The second group of participants involved ten purposively sampled representatives of *stakeholder* agencies who work in partnership with the service and/or support the same client group. They included day centre support workers, hostel key workers, community pharmacists, and healthcare professionals. The final group consisted of all four *staff* members directly involved in delivery of the service. Stakeholder and staff interviews were conducted to give insights into how the service was delivered ‘on the ground’ and what difference these actors considered it to have on patient experiences and outcomes. The interview topic guides employed for all three groups are provided (see Additional file 1).

All interviews were conducted on the premises of the service in which participants were recruited to the evaluation, which for people with experience of homelessness included four day centres/drop-ins, three homeless hostels, one B&B, and the city’s specialist homeless healthcare centre. All were conducted in private rooms with the exception of two patients whom were interviewed in quiet areas of public cafes after being enlisted from the street.^1^ None of the interviewees with experience of homelessness was known to researchers prior to data collection, but one PHOENIx staff member and one stakeholder interviewee were previously known to interviewers given their mutual involvement with national- or local-level homelessness and health policy forums. Almost all interviews were audio recorded; exceptions included the two patient interviews which were conducted in public cafes, and one stakeholder interview which was conducted in an area of the venue where confidentiality could not be guaranteed. All audio recorded interviews were transcribed verbatim^2^; detailed notes were recorded immediately after the others. A pseudonymised record of the number of interviewees conducted, demographic characteristics of interviewees, and key themes discussed were kept in the form of field notes so as to inform the sampling process and aid construction of the coding framework. Data were gathered over an 8 month period (August 2018 – March 2019), and saturation was deemed to have been reached before data collection ceased.

### Data analysis and ethics

Pseudonymised interview transcriptions and notes were analysed thematically, and in the case of individuals with experience of homelessness via framework analysis also, with the aid of NVivo qualitative data analysis software. A hierarchical coding frame based on the research questions, theoretically informed outcome domains, and initial themes drawn from preliminary transcript analysis was utilised in data analysis. Full coding of the entire dataset was conducted by one member of the research team (JB) and quality checked by the Principal Investigator (SJ). All names, where attributed to the quotations that follow, are pseudonyms. Descriptors identifying staff members or patients have been edited out of quotations, and specifics regarding homeless participants’ demographic and other characteristics have been presented in aggregate textual form only (see below) so as to preserve individual anonymity. No demographic or other potentially identifying details (such as role titles or professional credentials) are provided regarding staff and stakeholder interviewees for the same reason.

Participation was voluntary and written informed consent was obtained from all participants. Stakeholder and staff interviewees were invited to contribute by a member of the research team via email, at which time they were also sent a research information sheet providing details regarding the study aims, methods, and reporting protocols. Individuals with experience of homelessness were generally either approached directly by a member of the research team within outreach service settings or introduced to a researcher by PHOENIx’s voluntary sector street team worker. The latter strategy was invaluable in fostering contribution from patients who were wary of interacting with people unknown to them; it was also employed in initiating contact with those individuals recruited from the street. Almost all of the patients recruited to the study within homeless accommodation settings (hostels and B&B) were approached immediately after one of their consultations with a PHOENIx pharmacist. In all cases, people with experience of homelessness were told about the study, provided with an information sheet, given an opportunity to ask any questions about the evaluation, and invited to participate.^3^ Interviewees who were currently or recently homeless were given shopping vouchers as an incentive to counter non-response bias and as a gesture of thanks for their participation. Ethical approval for the study was obtained from the NHS Research Ethics Committee and Heriot-Watt University.

### Homeless participant characteristics

In terms of demographic characteristics, 33 of the interviewees with experience of homelessness were male and seven female. Their ages ranged between 19 and early 80s, with most in their 30s or 40s. With regard to their housing status at the point of interview, 15 were living in a homeless hostel, 11 had recently secured independent settled accommodation (but were still receiving support from homelessness services such as day centres), five were staying in a B&B, four were sleeping rough, two were staying temporarily with friends or relatives (sofa surfing), two were staying in a temporary furnished flat, and one lived in sheltered housing. The number and duration of homelessness episodes reported by interviewees varied considerably: one had become homeless for the first time within the 2 months prior to interview, whilst others reported having experienced homelessness intermittently throughout much of their adult lives. Their demographic and housing status profiles were thus broadly reflective of the sample of PHOENIx patients described in Lowrie F et al.’s recent analysis of the service’s patient records [23].

The interviewees with experience of homelessness self-reported a very wide range of (often long term) physical and/or mental health conditions, of varying degrees of severity. These included, amongst others: chronic obstructive pulmonary disease, diabetes, angina, arthritis, cirrhosis, HIV, osteoporosis, depression, anxiety, psychosis, and schizophrenia. Several described themselves as an alcoholic and many others reported drinking at harmful levels. A substantial number were past or current users of illicit drugs such as heroin, crack cocaine and/or New Psychoactive Substances. Some were polysubstance abusers, and/or consumed prescription drugs (e.g. Valium or diazepam) for non-medical purposes. The health conditions reported by interviewees, and their use of prescription medication, was also broadly reflective of the PHOENIx service’s patient profile as recorded by Lowrie F et al. (see above) [23].

## Results

The study’s findings can be summarised under three core themes, these including the influence of the service approach on: case finding and engagement; healthcare access and utilisation; and health-related outcomes.

### Case finding and engagement

There was a strong consensus among stakeholder interviewees that the service was highly effective at case finding and engaging with people experiencing homelessness who would otherwise ‘fall through the gaps’ of healthcare. They reported witnessing the staff engage successfully with many patients who had previously neglected their health seriously and/or failed to access healthcare (other than A&E) for prolonged periods of time.

*A lot of this client group would have access to [the HHS] … but for whatever reason quite often are not willing to engage with them … and if a lot of them are not registered with a GP or have other health problems … or generally they are just so chaotic that they’re not going to be going to get their prescriptions regularly, or they just don’t care about their health and so they’re just not making that effort to be getting their prescriptions... Then if [the service] team wasn’t there, then these people would be just falling through the cracks. They wouldn’t be engaging with anyone at all. (Stakeholder 6).*

Patient interviewees described their experience of interaction with the service in overwhelmingly positive terms. Many emphasised the positive influence that the friendly, informal and non-judgemental approach of staff had had on their willingness to engage. For some, pre-existing relationships with the voluntary sector street team worker facilitated introductions to and their trust in the pharmacists. Furthermore, the extended time spent in consultations, which exceeded that experienced in formal healthcare settings, and interest shown by the pharmacists in matters unrelated to health, meant that patients felt genuinely ‘listened to’ and respected:

*I have trouble addressing folk … I think my self-worth’s been knocked that much that I don’t think I’m worthy now, you know … [Name of pharmacist] is just approachable … and you get the impression when you’re speaking to them, they’re interested in you … They’re so nice and non-judgemental, and give off the impression that they genuinely want to help, which, in the medical profession is quite rare, because they’re so busy. (Geoff).*

*They’ve got a different approach. I’m not demeaning any of the health services’ people, doctors or nurses, but you’re more at ease with them [the pharmacist and street team worker] … My doctor’s a great guy in many ways, and the practice nurses and pharmacists, but it’s a different way of approach in here [day centre]. They make you more at ease … They spend time listening to you … It makes me feel good, aye. I feel really good, relaxed, the way they come across. (John).*

The convenience of the outreach clinics, combined with the immediacy of response (including provision of prescriptions where relevant), were also highlighted as key factors influencing initial engagement and retention:

*There’s days I don’t like going out, so I’d rather just jump down here [in B&B] and have a talk to them for half an hour, and get my tablets … I hate meeting people, and there’s times I don’t like going up [to HHS], so I usually miss dates, and my meds, and that, if I’m not going up and collecting it … Aye, I just jump down and get my prescription. (Isobel).*

*Whenever I go up to [the HHS] it’s an absolute nightmare half the time, because they only take, I think it’s eight or nine people … Where here [day centre] … You go in, you just sit and wait and then walk over … So for me, it’s convenient, it’s handy and I don’t have to worry about not getting an appointment. (Donald).*

Stakeholder interviewees also highlighted the service’s ‘stickiness’, that is, the pharmacists’ willingness to continue assessing and treating individuals after periods of disengagement, as an additional factor contributing to patient retention:

*I think they’re quite a sticky service … I think that when you look at some work around vulnerable people with complex issues or whatever, then they often do need a bit of a sticky service. They frequently didn’t turn up or didn’t do what was asked of them and you still stick with them. I can see evidence of that in terms of that they [the pharmacists] persevere with that. (Stakeholder 5).*

Together, these service characteristics fostered the generation of and/or enabled the capitalisation of ‘windows of opportunity’ when patients were engaged and motivated to address healthcare needs. This served to curtail the potential ‘leakage’ of patients whose intentions to access healthcare might have been genuine but whom were vulnerable to being ‘diverted’ by other people and/or competing priorities:

*See when you get somebody who’s sat there with COPD, can’t walk, and has to go to [the HHS], or somebody who’s got leg wounds which need to be treated there and then, but isn’t going to [the HHS], then [name of pharmacist] can do that. That’s such a difference, because once they’re out this building, we can lose them. The world that they exist in, that sort of chaotic and frenetic world that they exist in, drags them anywhere. (Stakeholder 1).*

*Signposting works for some people but not all, and people who it works least for are the people who are in the most chaos and for whom priority isn’t always health. If it’s not immediately accessible, that is to say where the person is at the time, and when the iron is hot at the time, so to speak … people don’t always make it to the place that’s been signposted. (Stakeholder 8).*

### Healthcare access and utilisation

The vast majority of interviewees with experience of homelessness reported having encountered barriers to accessing healthcare in the past, and these had deterred most from seeking support from or maintaining engagement with healthcare services. The informal and flexible outreach approach described above was reported to have been highly influential in mitigating these barriers. The ability to speak to a pharmacist in temporary accommodation, on the street, or in other services that they used regularly had for example alleviated some patients’ *anxiety associated with ‘going out’ and/or being in crowded places* such as clinic waiting rooms:

*I feel comfortable in this building. There’s not a lot of Glasgow city centre I feel comfortable in, including the walk up to [the HHS], because you meet every waif, stray, and bad-un on the way up there, and … you will bump into the wrong ones. (Graeme).*

*I can’t handle too busy places, so I scarper****.*** *(Campbell).*

On a related note, the service’s outreach element enabled patients to avoid other users of specialist homeless healthcare services, many of whom are (also) involved in substance misuse, given *perceived risks to their personal safety and/or recovery*:

*In [the HHS], I’ve got too many pals, or enemies … If it’s pals, they take me away for drink or drugs, and I end up steaming, or else I bump into somebody that I’ve hurt, or who’s hurt me. (Gary).*

The greater time available for consultations with the pharmacists overcame what some patients perceived as *insufficient time in standard GP appointments* for the comprehensive assessment of and response to healthcare needs:

*When I go in [to my GP] … He’s not got enough time. They’re on a time limit, aren’t they? They’re like, ten minutes with everybody, they’re just banging through everybody. It’s not really their fault, just … they’ve got that many people coming and going. (Malcolm).*

*They [GPs] deal with like one problem at a time, when you’ve got 15 problems. (Steven).*

A few interviewees reported that the service circumnavigated the issue of *limits to confidentiality when visiting specialist clinics* (e.g. for HIV), given that the designated timing of some such clinics are well known by members of the local homeless population thereby in effect (albeit unintentionally) ‘disclosing’ their diagnosis or condition:

*I think everybody knows what you’re up for [at HHS] on certain days … and I don’t want anybody to see me … Obviously, people see you coming down here [in B&B], but it’s not as if they know exactly what it’s for, do you know what I mean? … In here it’s a bit more confidential, because … they don’t know what you’re seeing [the pharmacist] for, so, aye, it’s all right. I prefer it, anyway. (Isla).*

The service also enabled some to proactively avoid mainstream healthcare settings where they had previously experienced *stigmatised attitudes* from staff or other patients:

*I was once sitting in a doctors’ surgery waiting to see a doctor with my wife, now ex, and someone else came in and the receptionist apologised to her for there being two drug users in the waiting room. In front of us! I couldn’t believe it. She [ex-wife] just went ballistic. (Ewan).*

In addition, by ‘taking the service to’ people experiencing homelessness, the influence of barriers associated with a *lack of motivation regarding and/or priority accorded to healthcare* are reduced:

*Sometimes I just can’t be arsed [going to HHS]. It’s a hell of a walk. And sometimes I bump into people on the way and don’t end up making it anyway [laughs]. There’s little risk of that happening when they [the pharmacists] come here [hostel]. (Peter).*

Finally, the provision of outreach also reduced barriers associated with *impaired mobility which limits access to healthcare services* which is a substantial problem for some individuals:

*It’s too far for them to go to [the HHS] in a wheelchair and stuff like that or with a Zimmer or any sort of walking aid. These guys can actually just come along here [in hostel] to see [name of pharmacist] … They get their needs met here … I think it brings a vital service to the folk that can’t get to the services. (Stakeholder 3).*

### Health-related outcomes

A definitive assessment of impacts on patient health would require a different methodology (ideally including a control group) and was thus beyond of the scope of this qualitative study, but patient interviewees did provide detailed narrative accounts of health-related outcomes that are worthy of comment. One, widely reported, was improved understanding of health-related conditions and the effects of medication, such as enhanced understanding of the impact of illicit drug use on long-term health, and the potential side effects of prescribed medication, to name but a few examples:

*They gave me a bit of stern advice. I didn’t actually realise that the likes of paracetamol can kill you. I didn’t actually realise that after - I think they said - 10 or 11 you can actually overdose from it. I was like, ‘Serious?’ I would take probably that on a day sometimes. They told me that and I’m like, ‘Right, okay. I didn’t realise that.’ (Donald).*

This outcome, and the informal and understanding approach of pharmacists described above, had alleviated the anxieties of many patient interviewees regarding their health and/or what should be expected if they were to be referred on to other (mainstream or specialist) healthcare services:

*They told me a wee bit, in layman’s terms, which the doctor’s never, ever sat down and told me … Aye, because the doctors … they’ll go like that, ‘Well, you’ve got cirrhosis of the liver’, and then they leave it like that … They just leave you there with that thought stuck in your head, and you’re like that, ‘Oh, shit, cirrhosis. Wait a minute, that’s dodgy. Am I going to die? Am I not going to die?’, all this. So, they [pharmacists] can answer a few of those questions... (Malcolm).*

Consultations with the pharmacists and voluntary sector street team worker, and in particular cognisance of the apparent ‘care’ and respect they expressed by taking time to converse with patients about matters unrelated to health, served to elevate consideration of health amongst patients’ many other (and often competing) priorities:

*They’ve encouraged me to give a toss, basically, about my wellbeing, you know, because I wasn’t really. I was getting really reckless and bang at it, as we say in the parlance of street drugs. I had a rotten habit, you know? … I would have continued on my merry way, and, there is only two ways out when you are a drug user. Death is one. (Geoff).*

Some patients also described experiencing direct and immediate impacts on health, such as having wounds dressed and/or receiving a new or modified prescription for medication which had a positive impact on their heath. For others, encouragement to access and/or onward referral to other healthcare services by the staff team had catalysed engagement with other primary or secondary healthcare provision.

*… my Prozac, they’re [GP surgery is] not really helping me … What [the pharmacist] suggested was … there’s a medicine out, it’s a two-in-one. It can help my blood pressure, and it can also help relax my muscles, or whatever … They went like that to me, ‘I’ll try and get in touch with them and try and get this organised for you, so you can go to the chemist’... (Sean).*

*They talked me into going on Methadone … [Otherwise] I would have continued losing weight and buying street junk, any, whatever poison it is these swine are putting in it, and, with the hectic lifestyle, you don’t eat enough as it is. I would have continued to deteriorate health-wise … My health has took a few steps better. (Geoff).*

Further to this, a number of stakeholders reported witnessing increased levels of patient adherence with prescription medication which they attributed to the contribution of the service:

*So if it wasn’t for the team issuing those prescriptions and seeing these patients regularly and doing the blood pressure checks and referring these people on, I don’t think anyone else would be able or willing to do it. So, the most kind of noticeable and tangible difference is … patients that weren’t taking their medicines before, or weren’t engaging with services are doing so because of them. (Stakeholder 6).*

## Discussion

This study’s findings lend further weight to the burgeoning evidence base on the effectiveness of flexible, person-centred and ‘sticky’ approaches when supporting severely and multiply disadvantaged homeless people [17, 19, 35, 36]. Most importantly, it highlights the substantial value of removing hurdles to access insofar as possible, by for example jettisoning predefined appointment times, eschewing conditionality requirements regarding attendance, and circumnavigating the need for patients to travel to formal medical clinics. Together, these actions increase medical practitioners’ capacity to catalyse and capitalise upon windows of opportunity, thereby increasing the potential for patients (and prospective patients) to engage with an intervention and experience health improvements. This finding has clear relevance for both health- and housing-focussed initiatives targeting this population, whilst also highlighting the utility of partnerships between organisations with expertise in both fields.

Resource implications notwithstanding [37], study findings also demonstrate the value of holistic approaches to health assessments of people experiencing homelessness. The greater time allowed for consultations and emphasis accorded to prevention and management of long-term conditions were welcomed by stakeholders and patients alike, not least because of the influence on engagement and retention levels. A number of stakeholder interviewees reported that the service complemented and relieved some of the pressure felt by other NHS provision, most notably by shouldering some of the responsibilities associated with the treatment of long-term conditions and filling gaps in healthcare for this patient group locally. Some stakeholder interviewees nevertheless highlighted a need for further clarity regarding where responsibility should lie for further diagnosis or treatment if an issue was picked up in an outreach setting but the patient was registered but not engaging with other healthcare services (including GP and/or addiction treatment) at the time.

It was notable that many patient and some stakeholder interviewees acknowledged that they did not fully comprehend the parameters of the pharmacists’ specialist expertise and prescription capabilities. In fact, whilst valuing their clinical competence, many patients confused the pharmacists with or mistook them for other healthcare professionals along gender-based lines (i.e. assuming females were nurses and males doctors) despite the great care taken by team members to be clear about their professional identities when interacting with patients and prospective patients. In the eyes of these interviewees, the service’s distinctiveness, and greatest value, lay not so much in the clinicians’ professional identities or roles per se, but in the approach employed. What ‘worked’ for them was the service’s relationality, ‘stickiness’, and actions taken to do everything possible in the ‘here and now’. There are clear lessons that can be drawn here for the delivery of healthcare to this population more generally, regardless of practitioners’ clinical backgrounds and identities.

The study findings also suggest that specialist clinics targeting homeless people are not immune from some of the criticisms typically directed at mainstream healthcare services [13–15]. Notably, whilst specialist clinics aim to offer a degree of protection from the stigma reported by many homeless people in mainstream medical centres, some individuals will nevertheless actively avoid these centres given fears for their personal safety and/or the risks that encounters with other members of the homeless population may pose to their recovery from substance misuse and/or mental health problems. For this reason, further thought might valuably be given to the most effective means of handling the processes and politics of clinic waiting rooms. In a similar vein, it seems that more might be done to preserve the confidentiality of people attending clinics for specific conditions (e.g. blood borne viruses) at designated times when these are well known to members of the local homeless population.

Finally, reflecting on implications for public health more generally, it was evident that the pharmacy outreach team uncovered a substantial prevalence of hidden chronic disease, such as diabetes, cardiovascular disease, respiratory problems and hypertension amongst patients. Traditionally the foregrounding of mental health and harmful substance use in the public health literature has obscured these chronic health issues from view [38, 39]. Nonetheless, as members of the homeless population age, chronic disease is being uncovered as an important but neglected health issue [40–42]. Efforts are urgently needed to prevent the rise in cardiovascular disease and diabetes for people experiencing homelessness and to increase access to health care for those who already have chronic disease.

## Conclusions

By investigating patient and other stakeholder experiences and perspectives of the PHOENIx service, this qualitative study indicates that clinical prescribing outreach delivered within homelessness services and on the street can be highly effective at case finding and engaging with homeless people who would otherwise ‘fall through the gaps’ of existing healthcare. Specifically, the outreach element operates as a bridge to both primary and secondary healthcare for people experiencing homelessness who are reluctant to utilise or physically unable to access alternative (mainstream or specialist ‘homeless’) provision. The informal, flexible and person-centred approach, and proactive immediacy of response (including provision of prescriptions where relevant), appear to be critical ingredients in the initiation and maintenance of patient engagement by enabling practitioners to catalyse and/or capitalise on windows of opportunity when patients are motivated to address healthcare needs. Together, these characteristics assist patients to overcome many of the barriers to healthcare commonly faced by people experiencing homelessness. Whilst further research is needed to quantify the nature and extent of impacts on specific health outcomes, these qualitative findings provide ground for substantial optimism regarding the potential of such an approach to contribute to better health for members of this extremely vulnerable population.

## Supplementary Information

**Additional file 1.** Topic guides for interviews with staff, stakeholders, and people with experience of homelessness

## Data Availability

The qualitative datasets generated and analysed during the current study are not publicly available given the vulnerability of many of the interviewees, the sensitivity of issues covered, and assurances granted to participants regarding confidentiality and anonymity. The interview topic guides are provided as an Additional file. Participant information sheets are available upon request, as is a copy of the health check tool used by the PHOENIx service pharmacists.

## Footnotes

1 Questions were carefully selected in these latter instances so as to ensure that interviewees were not asked and/or did not feel compelled to disclose personal or sensitive information when confidentiality could not be guaranteed.

2 A sample of transcriptions was quality checked by interviewers. Whilst any requests from participants to check or comment on the transcription from their interview would have been respected, interviewees were not specifically asked to do so on ethical and data protection grounds, especially the imperatives to minimise burden on participants and mitigate against breaches of confidentiality. Resource restrictions and the challenges involved in re-contacting homeless people meant that the research team was unable to invite feedback on preliminary findings in any systematic way. A report of findings was however made available to all agencies supporting the study that provided services to interviewees with experience of homelessness.

3 A small minority of people with experience of homelessness who were invited to contribute declined to do so. The number of refusals and reasons for declining were not recorded.
