# Supplementary material for: Outreach-based clinical pharmacist prescribing input into the healthcare of people experiencing homelessness: a qualitative investigation
Source: BMC Health Serv Res. 2021 Jan 4;21:7. doi: 10.1186/s12913-020-06013-8 (PMC7780619; doi:10.1186/s12913-020-06013-8)
Supplement: Supplementary file 1 — Additional file 1. Topic guides for interviews with staff, stakeholders, and people with experience of homelessness [file 12913_2020_6013_MOESM1_ESM.pdf]

ADDITIONAL FILE 1

INTERVIEW TOPIC GUIDES

(NB: names of organisations and individuals have been redacted to preserve anonymity)

Qualitative Study of Clinical Pharmacist Input into the Care of People Experiencing Homelessness

Staff Interview Topic Guide

Preamble:

- Reiterate purpose of evaluation
- Remind re voluntary participation and confidentiality
- Describe when findings reported and how may be accessed
- Ask permission to record interview
- Any questions?

What is your current job title or role?

Can you talk me through:

- How the service identifies and approaches potential patients
- What is involved in the assessment process
- How any identified needs are responded to

What if anything would you consider to be distinctive or unique about the way that you and your colleagues work with patients (as compared with doctors and nurses at [name of specialist homeless health service] or other pharmacists who might work with them elsewhere)? [Probe fully re differences in what is done, how it is done etc.)

What do you consider to be the main advantages and disadvantages of delivering the service outwith [name of specialist homeless health service] (in hostels, [names of other services], on street etc.)?

In your view, how effective has the service been at:

- Identifying and making contact with homeless people who may benefit from pharmacist support? [Prompt: how well known is it amongst homeless people?]
- Engaging (and maintaining engagement with) these individuals? [prompt: how receptive are potential users to it?]

What (if anything) might be done to improve potential patients' uptake of the service?

In your view, how effective has the service been at improving users'…:

- health outcomes? [probe re physical, mental, substance misuse]
- other (housing, etc.) outcomes?
- willingness to use/engage with other services?

What would you consider to be the service's greatest…:

- Strengths? (probe re approach, locations etc.)
- Weaknesses? (probe as above)

What would you consider to be the main challenges that you face when working on a day-to-day basis? To what extent and how are these overcome? [probe re service user characteristics/ engagement, nature of their health issues, availability/quality of other services, policy context etc.]

Have any 'lessons been learned' by the service to date? What, if so? [probe re policies/procedures, intensity/timing of support, modes of delivery etc.] Which of these have been or should be acted upon?

Do you think the service is more effective at working with some groups, or individuals with particular characteristics, than others? Who and why? (probe re age, gender, nationality/migration status, type of health issue, type/severity of substance misuse, willingness to engage etc.)

How well does the service work with other service providers locally? Why is that?

Do you think the service could be improved? How, if so?

[Describe planned RCT]. In your view:

- What is the best way of encouraging people experiencing homelessness to participate in the study? (probe re how/where to advertise, who should approach them, incentives etc.]
- What outcomes do you think should be measured in the study? [probe re health and other outcomes, hard and soft indicators]

Is there anything else that I should have asked you, or that you would like to add?

Thanks and close

Qualitative Study of Clinical Pharmacist Input into the Care of People Experiencing Homelessness

Stakeholder Interview Topic Guide

Preamble:

- Reiterate purpose of evaluation
- Remind re voluntary participation and confidentiality
- Describe when findings reported and how may be accessed
- Ask permission to record interview
- Any questions?

What is your current job title or role?

What involvement have you (or your agency) had with the specialist pharmacy input service? [probe re nature/length of involvement]

What if anything would you consider to be distinctive or unique about the way that the pharmacy service works with patients (as compared with doctors and nurses at [name of specialist homeless health service] or other pharmacists who might work with them elsewhere)? [Probe fully re differences in what is done, how it is done etc.)

What do you consider to be the main advantages and disadvantages of delivering the service outwith [name of specialist homeless health service] (in hostels, [names of other services], on street etc.)?

In your view, how effective has the service been at:

- Identifying and making contact with homeless people who may benefit from pharmacist support? [Prompt: how well known is it amongst homeless people?]
- Engaging (and maintaining engagement with) these individuals?
- Improving health outcomes? [probe re physical, mental, substance misuse]
- Improving other (housing, etc.) outcomes?

What would you consider to be the service's greatest…:

- Strengths? (probe re approach, delivery, location etc.)
- Weaknesses? (probe as above)

What (if any) 'difference' does the service make to the experiences of and outcomes for its users? How and why?

Do you think the service is more effective at working with some groups, or individuals with particular characteristics, than others? Who and why? (probe re age, gender, nationality/migration status, type/severity of substance misuse, type of health issue, willingness to engage etc.)

Do you think the service could be improved at all? How, if so?

[Describe planned RCT]. In your view:

- What is the best way of encouraging homeless people to participate in the study? {probe re how/where to advertise, who should approach them, incentives etc.]
- What outcomes do you think should be measured in the study? [probe re health and other outcomes, hard and soft indicators]

Is there anything else that I should have asked you, or that you would like to add?

Thanks and close

Qualitative Study of Clinical Pharmacist Input into the Care of People Experiencing Homelessness

Service User / Non-User Topic Guide

Preamble:

- Reiterate purpose of evaluation
- Remind re voluntary participation and confidentiality
- Describe when findings reported and how may be accessed
- Ask permission to record interview
- Any questions?

Background

Can you please tell me a little bit about yourself to begin with…

Housing circumstances/histories:

- Where are you staying at the moment? [probe homelessness/housing status]
- How long have you been staying there? [probe for length of current homelessness episode]
- Have you ever been homeless before? How long ago / how many times if so? [probe to get sense of approx duration/number of episodes of homelessness]

Demographic and health characteristics:

- Do you mind my asking how old you are?
- How would you describe your health just now? [probe: very bad, bad, ok, good, very good]
- Do you have any current health-related problems? [Probe to develop a sense of]:
    - Physical health issues
    - Mental health issues
    - Substance misuse issues [probe: type, severity]

Service use *[Ask of people recruited on the basis of having used the service. Begin by checking that they understand which service we are evaluating. Note that members of the pharmacist team include: [names of pharmacists]. Locations that they work include: [names of relevant services].*

How did you first come into contact with the pharmacy service? Where were you when you were first approached and asked if you wanted to have health check and medicines review by a member of the team ([pharmacist names])?: [Probe: [names of relevant services], street etc.]

Had you heard about it before that? If so, what (if anything) did you know about it? How did you first hear about it?

How well known is the service amongst homeless people in Glasgow?

Can you remember what your first impressions of it were?

What did you think of the questions that the pharmacist asked you, and the way they were asked?

How many times have you used the service / met with the staff?

Please talk me through what happens when you meet with one of the pharmacists ([names of pharmacists])?: [probe: where do you meet, what do you talk about, how long do conversations take, what happens afterwards etc.]

Is the service 'different' or 'distinctive' from others in any way? How, if so? [probe: type of support, attitude of staff, length of consultations, contacts following no-shows for appointments, support attending other appointments etc.]

If have been in contact with pharmacist service outwith [name of specialist homeless health service]: What if any difference does having your health assessed at [name of relevant service(s)] (rather than going to appointments at [name of specialist homeless healthcare service]) make? What are the benefits and/or disadvantages of having the service 'come to you'? [Probe fully re reasons for preferences – distance, appointment restrictions, waiting time, rules, risk of bumping into people they'd rather not encounter, approach of staff etc.)

What if any difference does dealing with a pharmacist rather than a doctor or nurse make? Do they do or talk about different things? Or do/talk about them in a different way? Who do you prefer to meet with and why?

What things do you like most / least about the pharmacy service?

What (if any) impact has the service had on your:

- health [probe mental and physical]
- use of prescription medication
- use of other services? [probe health and on-health-related]
- [other outcomes?]

Could anything be done to improve the service? What, if so?

What might be done to increase uptake of the service by other homeless people?

Service non-use *[Ask of people recruited on the basis of NOT having used the service.]*

Begin by describing the service briefly (see note to interviewer above) and checking that they have not used it

Have you heard of that service?

- *If so*, what is your understanding of what is provided? *If not,* how might it be better advertised? [probe: where, how, when, by whom?]

Why have you not used it to date? [probe: lack of awareness or other reasons]

What might encourage you to consider using such a service? [probe fully: type of support, conditions of access/use, manner of delivery, location]

What would you hope to get out of using such a service?

Proposed RCT *[Ask of all participants]*

The pharmacists and their employers, NHS Greater Glasgow and Clyde, are looking to find out what if any difference the pharmacist input makes to people's health by doing another, bigger study after this one. That study will involve a very large number (hundreds!) of people and assess potential effects on a wide range of aspects of their health. We would be really grateful if could share your thoughts on what the study should measure and how people who are homelessness might most effectively be encouraged to be involved.

What do you think would be the best way to encourage people who are homeless to participate in the study? [probe re how/where to advertise, who should approach them, where they could be approached, incentives etc.]

What outcomes do you think should be measured in the study? [probe re heath and other outcomes, hard and soft indicators, Referral to other services e.g. addictions, podiatry, dental health support worker…]

Close

Is there anything else that I should have asked you, or that you would like to add?

Thanks and close
